# Reasons for women aged 50 years and older to seek gynaecological advice and treatment

**DOI:** 10.25646/6065

**Published:** 2020-06-30

**Authors:** Laura Krause, Lorena Dini, Franziska Prütz

**Affiliations:** 1 Robert Koch Institute, Berlin Department of Epidemiology and Health Monitoring; 2 Charité – Universitätsmedizin Berlin Institute of General Practice

**Keywords:** GYNAECOLOGY, REASONS TO SEEK TREATMENT, OUTPATIENT CARE, OLDER WOMEN, GERMANY

## Abstract

Gynaecological care is generally perceived as focused on reproductive health. However, when women enter the non-reproductive life phase, other reasons to seek gynaecological care gain in importance. This paper presents findings on the reasons for women in the 50 years and older age group to seek gynaecological consultation and treatment. Our findings are based on data from the German Health Interview and Examination Survey for Adults (DEGS1, 2008–2011), conducted by the Robert Koch Institute (RKI), as well as the 2016 claims data from the Associations of Statutory Health Insurance Physicians (KVen), provided by the Central Research Institute of Ambulatory Health Care in Germany (Zi). At this age, cancer screening and menopausal complaints can become, as DEGS1 data shows, important reasons to seek gynaecological services. Around 65.0% of 50- to 79-year-old women took advantage of breast palpation examinations during the last twelve months, and 58.0% underwent cervical cell smear tests (pap smear). 47.2% of women had their last menstrual period at age 50 or later. KV data shows that with 45.3% and 33.1% of cases respectively, menopausal symptoms (International Statistical Classification of Diseases and Related Health Problems, 10th revision, ICD-10: N95) and screening for malignant neoplasms (ICD 10: Z12) were the most frequently billed services. The data clearly shows reasons for consultation and treatment of women aged 50 years and older and these should therefore be considered in treatment planning and design.

## 1. Introduction

The public and research community primarily relate gynaecological care to reproductive health and questions such as family planning, pregnancy and birth [1]. As women near the end of their reproductive years, new reasons to seek consultation and treatment with outpatient gynaecological service providers arise, in part due to hormonal changes that occur during menopause [2–4]. Breast and endometrial cancer incidence rates increase with age and, following menopause, conditions such as urinary incontinence, osteoporosis and uterine prolapse become more frequent [5, 6]. Some conditions may necessitate surgery that requires outpatient medical aftercare [7–9]. From the perspective of prevention, cancer early detection gains great importance in gynaecological practice [10]. For women of specified age groups, statutory health insurance offers cancer screening tests as a standard benefit. In January 2020, early detection of cervical cancer was changed from an opportunistic screening offer to an organized screening programme, including an annual cervical cell smear (20- to 34-year-old women) or a cervical cell smear and human papilloma virus test every three years (age 35 and older). For the early detection of breast cancer, women aged 30 years and older are offered annual breast palpation examinations, and 50- to 69-year-old women can undergo a mammography of both breasts within the scope of a biennial mammography screening [11]. Some patients will request further consultation on mammography screening by their gynaecologist [12].

**DEGS1**

**Data holder:** Robert Koch Institute

**Objectives:** To provide reliable information about the population’s health status, health-related behaviour and health care in Germany including analysis of temporal developments and trends.

**Survey method:** Questionnaires, physical examinations and tests, a physician interview, a medication interview and laboratory investigations (blood and urine sample).

**Population:** German resident population, aged 18 and above

**Sampling:** Registry office sample; randomly selected individuals from 180 communities in Germany were invited to participate (120 original sample points of the German National Health Interview and Examination Survey 1998 and 60 new sample points).

**Participants:** N=8,151 (4,283 women; 3,868 men). The sample included persons who were newly recruited and those who had already participated in the German National Health Interview and Examination Survey 1998 (mixed design).

**Response rate:** 62% among revisiting participants and 42% first time participants

**Survey period:** 2008 to 2011

**Data protection:** DEGS1 is subject to strict compliance with the data protection regulations of the Federal Data Protection Act and has been approved by the Federal Commissioner for Data Protection and Freedom of Information in Germany. Charité – Universitätsmedizin Berlin’s ethics committee assessed the ethics of the DEGS1 study and provided its approval (No.EA2/047/08). Participation in DEGS1 was voluntary. The participants were informed about the aims and contents of the studies and about data protection. Informed consent was obtained in writing.

More information in German is available at www.degs-studie.de

With regard to the provision of gynaecological services, the health of women aged 50 years and older is not the only factor to be considered, as the overall uptake of gynaecological services also considerably decreases with age (see Focus Article Gynaecology and general practitioner services utilisation by women in the age group 50 years and older in this issue of the Journal of Health Monitoring). Demographic ageing, moreover, is leading to an increasing number of older women (see Fact Sheet Demographics of the female population aged 50 years and older in Germany’s north east region – Selected aspects, also in this issue of the Journal of Health Monitoring). Against this backdrop, and due to the increasing difficulties related to ensuring outpatient care in rural regions, the project ‘Frauen 5.0’ (Regionale Versorgung von Frauen über 49 Jahre durch Fachärztinnen und Fachärzte für Gynäkologie und für Allgemeinmedizin) was developed, which is funded by the Innovation Fund of the Federal Joint Committee [13]. The project analyses the provision of gynaecological and general practitioner (GP) services to women aged 50 years and older in Germany’s north east region (Berlin, Brandenburg and Mecklenburg-Western Pomerania). The project aims to gain an overview of the levels of care that are provided and, building on this, develop concepts to secure outpatient gynaecological care for this group within the population.

This paper describes gynaecological conditions that affect women aged 50 years and older over time, and possible reasons for gynaecological appointments. The results are based on data from the German Health Interview and Examination Survey for Adults (DEGS1, 2008–2011), conducted by the Robert Koch Institute (RKI). This data allows us to determine the possible reasons why women in this group sought gynaecological consultation and treatment. Furthermore, data from the Associations of Statutory Health Insurance Physicians (KVen), provided by the Central Research Institute of Ambulatory Health Care in Germany (Zi), reveals the most frequent diagnoses in gynaecological practices in 2016.

## 2. Methodology

### 2.1 Sample design and study implementation

As no representative data exists for Germany that indicates which gynaecological symptoms or need for consultation and treatment lead to an uptake of the services provided by gynaecological practices, this paper builds on multiple data sources that allow us to approximately describe the situation.

Between 2008 and 2011, as part of DEGS1, the RKI collected representative data on the health of the German population aged 18 to 79 years. This study, which covered 8,151 participants, included questionnaires, interviews, physical examinations and tests. The DEGS1 concept and survey design have previously been described in detail [14, 15]. DEGS1 included questions on gynaecological conditions and symptoms which could lead women to seek gynaecological services [5–10]. We can group these reasons into the following four categories: ‘gynaecological cancer screening’, ‘menopause and contraception’, ‘diseases and complaints’ and ‘gynaecological operations’. These are thereby only reasons for which women potentially sought gynaecology services; the survey did not ask whether women then actually visited a gynaecologist.

Claims data from the KVen, provided by the Zi (2016) provide further indicators of actual service provision and uptake. Every three months, the KVen provide the Zi with pseudonymised claims data from the quarterly accounts of office-based physicians. The information on the treatment cases, which this data contains, makes it possible to precisely record the coded diagnoses [16]. Therefore this data permits an assessment of actually provided gynaecology services in outpatient practices either across Germany or for certain regions. Based on a special evaluation on request of the RKI, this paper presents the ten most frequently recorded codes from the 10th revision of the International Statistical Classification of Diseases and Related Health Problems (ICD-10, three-digit codes) in gynaecology practices for women aged 18 years and older in Germany. Furthermore, we report the 20 most frequently recorded ICD-10 codes (three digits) for women aged 50 years and older in gynaecology practices in the north east region (Berlin, Brandenburg and Mecklenburg-Western Pomerania). We also present data from GP practices that refer to gynaecological diagnoses.

### 2.2 Statistical methods

On the basis of data from DEGS1 (2008–2011), we initially determine gynaecological conditions and operations in 18- to 79-year-old women (n=4,198). We also report potential reasons to seek gynaecological consultation and treatment by women aged 50 years and older (n=2,287) and provide these as prevalences (in percent). DEGS1 calculations were carried out using a weighting factor that corrects deviations within the sample from the population structure (as of 31 December 2010) with regard to age, sex, region, citizenship, type of municipality and education [14].

Based on the 2016 KV claims data that we received from the Zi, the most frequent ICD-10 diagnoses (three-digit codes) in gynaecology and GP practices are presented as the number of cases and proportion (in percent) of all cases.

## 3. Results

The prevalence of conditions and symptoms that women receive treatment for in gynaecology practices increases with age (Table 1). They include urinary incontinence, osteoporosis, uterine prolapse, as well as breast cancer and gynaecological cancers such as cervical, endometrium and ovarian cancers. The proportion of women who undergo gynaecological surgeries, such as hysterectomy and ovariectomy, also, as expected, increases with age.

**Figure 1:**
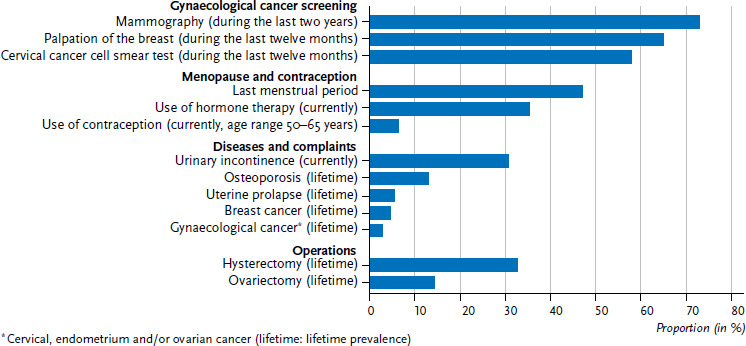
Reasons for 50- to 79-year-old women to seek consultation and treatment at gynaecology practices (n=2,287) Source: DEGS1 (2008–2011)

Figure 1 presents the prevalences for these conditions as recorded in DEGS1 data for the group of 50- to 79-year-old women, as well as further possible reasons to seek an appointment at a gynaecology practice. Cancer screening, in particular, plays an important role: 73.1% of women had undergone a mammography during the last two years (examinations carried out as part of the mammography screening programme and during normal practice). 65.0% had undergone breast palpation during the last twelve months and 58.0% cervical cancer screening (cervical cell smear test, pap smear).

Menopause is a further reason to seek consultation and treatment. Nearly half of all women (47.2%) had their last menstruation at the age of 50 or later (average age for the last menstrual period in Germany: 49.7 years [17]). Over one third of women (35.5%) stated that they used menopausal hormone therapy.

Gynaecological conditions and post-operative care can also lead women to seek an appointment at gynaecology practices: at the point of survey, 30.8% of women stated they had acute urinary incontinence, and around one third (32.7%) had undergone hysterectomy. Osteoporosis (with a lifetime prevalence among 50- to 79-year-old women of 13.1%), uterine prolapse (5.5%), breast cancer (4.7%), as well as gynaecological cancers (cervical, endometrium, ovarian cancer 2.9%) could be further reasons to seek gynaecological treatment.

6.5% of 50- to 65-year-old women said they used contraceptives. Of these, 22.9% used condoms, 46.6% used a contraceptive coil and 27.9% said they used the pill (data not shown). However, due to the low number of cases (n=80), these results should be interpreted with caution. Nonetheless, they do show that a need for consultation and, in some cases, medical check-up examinations may also exist here [18–20].

An analysis of the most frequent diagnoses billed by gynaecology practices for women aged 18 years and older showed that menopausal and climacteric states (ICD-10: N95) ranked fourth (Table 2). The diagnoses ranked second and third (N89: other specified noninflammatory disorders of vagina, Z12 encounter for screening for malignant neoplasms) are, as the following analyses show, also frequently billed for women aged 50 years and older.

**Figure 2:**
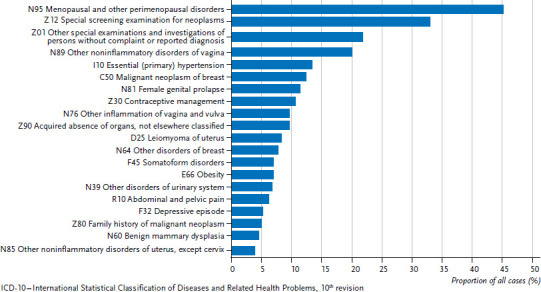
The 20 most frequent ICD-10 codes (three digit) recorded by gynaecological practices for women aged 50 years and older in the region north east (Berlin, Brandenburg and Mecklenburg-Western Pommerania) (n=1,448,162) Source: Central Research Institute of Ambulatory Health Care in Germany (2016) [21]

Figure 2 presents the 20 most frequently billed diagnoses in gynaecology practices for women aged 50 years and older in the north east region in 2016. The largest recorded proportion, 45.3% or 655,459 cases, concerned menopausal states (ICD-10: N95). Second came encounters for screening for malignant neoplasms (Z12: 478,683 cases, 33.1%). Other special examinations (Z01: 316,622 cases, 21.9%), which also include general gynaecological examinations, ranked third. Further frequently billed diagnoses were noninflammatory disorders of vagina (N89: 291,281 cases, 20.1%) and other inflammations of the external genitals such as vaginitis and vulvitis (N76: 141,013 cases, 9.7%), but also hypertension (I10: 194,762, 13.4%), breast cancer (mammary carcinoma, C50: 178,947, 12.4%), uterine prolapse (N81: 164.941, 11.4%), contraception (Z30: 155,573, 10.7%) as well as acquired absence of organs (Z90: 140,106, 9.7%). One possible reason for the frequency of breast cancer as a billing diagnosis could be, considering the relatively early age at the onset of disease and the often good prognosis, regular aftercare.

Gynaecology practices not only bill gynaecological diagnoses but also internal medicine diagnoses such as hypertension (I10) and obesity (E66). Further diagnoses included conditions with a psychological/psychiatric background, such as somatoform disorders (F45) and depressive episodes (F32), as well as unspecified diagnoses (Z01, Z12, Z90).

The results for the 20 most frequent diagnoses coded by gynaecological practices for women aged 50 years and older are widely congruent between the north east region and Germany as a whole, whereby the order of these conditions sometimes differs slightly (data not shown). The 20 most frequent diagnoses coded for women aged 50 years and older by GPs in the region north east and for Germany as a whole include no gynaecology-related diagnoses (data not shown).

## 4. Discussion

The results highlight that certain gynaecological conditions and operations occur more frequently with age, and give an overview of reasons why women aged 50 years and older might seek gynaecological consultation and treatment. DEGS1 data (2008–2011) and KV data (2016) both indicated that cancer screening and menopause were the most frequent reasons for appointments at gynaecology practices. Inflammatory and noninflammatory disorders of the external genitals, uterine prolapse, as well as breast cancer and gynaecological cancers were among the further identified key reasons to seek consultation and treatment. Gynaecology practices also billed some typical GP diagnoses, such as hypertension and obesity. GPs, however, very rarely diagnose gynaecology-related conditions.

Prevalence estimates that could be compared to DEGS1 data are rare. According to international studies, the average age for the onset of menopause is around 51 years [22, 23]. Data from the Techniker Krankenkasse (TK) health insurance on menopausal hormone therapy shows that in 2017, 6.6% of the 45- to 65-year-old women covered by the TK were prescribed hormone therapy during menopause. The figures for 2010 and 2000 were 9.6% and 37.0%, respectively [24]. DEGS1 data shows considerably higher figures for the proportion of women who use hormone therapy during menopause (35.5% between 2008 and 2011), but this data refers to a different age range and includes policy holders of all insurers. International studies estimate a uterine prolapse prevalence of around 30% [25–27]. However, these results are based on gynaecological examination data; how many of these women actually had symptoms was not considered in the studies. Current prevalences of cancer are shown in the report 'Cancer in Germany' of the Center for Cancer Registry Data and the Association of Population-based Cancer Registries in Germany e.V. [28]. Comparisons between DEGS1 data and cancer registry data show differences in the disease spectrum and in the average age of cancer diagnosis. This indicates that the survey participants are not representative of women with a cancer diagnosis in Germany in general. Women with severe courses of disease are likely to be underrepresented. Also, the number of cases (Table 1) is too small to achieve the – actually desirable – differentiation to validly map different types of cancer according to age.

Representative studies by the Federal Centre for Health Education (BZgA) on use of contraception showed that in Germany in 2018, 39% of 40- to 49-year-old women took the pill, 34% used condoms and 20% a contraceptive coil (2011: 34%, 26% and 13%, respectively) [20]. One in two women that used some kind of contraception stated that questions on contraception had led to an appointment in gynaecology practices [20]. The study did not examine contraceptive behaviour of women aged 50 years and older. BZgA studies show gynaecologists to be important contact persons for women’s health-related questions, yet the (potential) reasons to seek consultation and treatment in gynaecology practices can obviously only represent a proportion of all female health concerns. Due to be published this year, the women’s health report of Federal Health Reporting will contain a comprehensive overview of the diverse aspects of women’s health [29].

Both survey and claims data have their limitations. While DEGS1 is generally representative for the German population, the survey tends to underrepresent people living in institutions, as well as older and less healthy people, an aspect which an analysis concerned with middle-aged and older women should take into account. Health conditions are self-reported in the survey, which could lead to a certain level of recall bias. Events such as a hysterectomy, however, are remembered with a high degree of reliability [30]. In addition, DEGS1 did not ask women whether health problems actually led them to seek outpatient gynaecology services. A strength of claims data is the large sample size and that it comprises all women covered by statutory health insurance. However, the data is collected for billing purposes, and it is therefore impossible to directly deduce the prevalences for particular conditions in the population. Moreover, since the introduction of lump sum payment in 2008, data is only available for cases per quarter and practice [31].

Women are in the reproductive phase for less than half of their entire lifespan. A perception of women’s health as being primarily reproductive health does not do justice to this fact and veils the consultation and treatment needs of women aged 50 years and older. Taking these needs into account does not imply postulating new reasons why women should seek treatment (in the sense of a medicalisation of life phases [32]), but should, rather, contribute to ensuring that middle-aged and older women also receive adequate care, for example regarding cancer screening examinations. Against this backdrop, we should mention two further findings: the decreasing number of women who seek appointments with gynaecologists with age – around 80% of 18- to 29-year-olds, but only 60% of 50- to 79-year-old women have at least one appointment per year in a gynaecology practice – as well as the increasing number of older women due to demographic ageing and the frequently higher average age found in rural regions [33, 34]. When planning care, it is important to consider that the screening programmes for the ealy detection of breast cancer and – since January 2020 – cervical cancer have an influence on the use of gynaecological services, as have the decreasing number of women who use hormone therapy during menopause [24] and the declining number of hysterectomies [35]. Reasons for seeking gynaecological services from the perspective of women themselves were analysed by the project ‘Frauen 5.0’, as were concepts to ensure that women in the 50 years and older age group receive adequate gynaecological services, in particular in sparsely populated rural regions, and regional models that include the relevant actors were developed [1].

## Key statements

- Cancer screening is one of the main reasons for women aged 50 years and older to seek outpatient gynaecological services.
- Menopausal symptoms also frequently lead women to seek gynaecological services; nearly half of all women aged 50 continue to menstruate.
- Women aged 50 years and older are an important group of gynaecological patients.

## Figures and Tables

**Table 1:** Prevalence of specific diseases and operations in 18- to 79-year-old women by age (n=4,198) Source: DEGS1 (2008–2011)

	Age group					
	18-29 years	30-39 years	40–49 years	50-59 years	60-69 years	70-79 years
**Diseases**						
Urinary incontinence (currently, n=3,276)	4.2%	11.4%	17.1%	23.2%	30.1%	42.3%
Osteoporosis (lifetime, n=313)	n.s.	n.s.	n.s.	4.1%	12.7%	25.2%
Uterus prolapse (lifetime, n=136)	0.2%	0.0%	2.5%	4.7%	4.6%	7.6%
Breast cancer (lifetime, n=112)	0.0%	0.1%	1.3%	2.9%	5.4%	6.2%
Gynaecological cancers (lifetime, n=87)	0.2%	0.9%	1.1%	3.3%	3.6%	1.7%
**Operations**						
Hysterectomy (lifetime, n=784)	0.0%	0.5%	10.2%	26.8%	35.2%	38.0%
Ovariectomy (lifetime, n=349)	0.0%	1.1%	4.5%	9.2%	15.8%	19.5%

n.s. = not surveyed

**Table 2:** The ten most frequent ICD-10 codes (three digits) used by gynaecological practices for women aged 18 years and older in Germany Source: Central Research Institute of Ambulatory Health Care in Germany (2016)^*^ [21]

Rank	ICD-10 Code	Diagnosis	Number of cases	Proportion
1	Z30^**^	Contraceptive management	18,734,546	39.15%
2	N89	Other noninflammatory disorders of vagina	12,931,229	27.0%
3	Z12^**^	Special screening examination for neoplasms	11,447,844	23.9%
4	N95	Menopausal and other perimenopausal disorders	7,036,573	14.7%
5	Z01^**^	Other special examinations and investigations of persons without complaint or reported diagnosis	6,418,212	13.4%
6	N76	Other inflammation of vagina and vulva	4,835,146	10.1%
7	N94	Pain and other conditions associated with female genital organs and menstrual cycle	4,410,731	9.2%
8	N92	Excessive, frequent and irregular menstruation	3,883,673	8.1%
9	R10	Abdominal and pelvic pain	3,122,270	6.5%
10	C50	Malignant neoplasm of breast	2,063,227	4.3%

ICD-10 = International Statistical Classification of Diseases and Related Health Problems, 10^th^ revision

^*^ Quarterly collection of case numbers, multiple registration of responses possible

^**^ Z diagnoses describe factors influencing health status and contact with health services.
